# Tensile and Compressive Responses of Ceramic and Metallic Nanoparticle Reinforced Mg Composites

**DOI:** 10.3390/ma6051826

**Published:** 2013-05-07

**Authors:** Khin Sandar Tun, Wai Leong Eugene Wong, Quy Bau Nguyen, Manoj Gupta

**Affiliations:** 1 Department of Mechanical Engineering, National University of Singapore, 9 Engineering Drive 1, 117576, Singapore; E-Mails: sandar_k_tun@ite.edu.sg (K.S.T.); mpenqb@nus.edu.sg (Q.B.N.); 2 School of Mechanical and Systems Engineering, Newcastle University International Singapore (NUIS), 180 Ang Mo Kio Ave 8, 569830, Singapore; E-Mail: eugene.wong@ncl.ac.uk

**Keywords:** metal matrix composites, microwave sintering, mechanical properties, microstructure, scanning electron microscopy, X-ray diffraction

## Abstract

In the present study, room temperature mechanical properties of pure magnesium, Mg/ZrO_2_ and Mg/(ZrO_2_ + Cu) composites with various compositions are investigated. Results revealed that the use of hybrid (ZrO_2_ + Cu) reinforcements in Mg led to enhanced mechanical properties when compared to that of single reinforcement (ZrO_2_). Marginal reduction in mechanical properties of Mg/ZrO_2_ composites were observed mainly due to clustering of ZrO_2_ particles in Mg matrix and lack of matrix grain refinement. Addition of hybrid reinforcements led to grain size reduction and uniform distribution of hybrid reinforcements, globally and locally, in the hybrid composites. Macro- and micro- hardness, tensile strengths and compressive strengths were all significantly increased in the hybrid composites. With respect to unreinforced magnesium, failure strain was almost unchanged under tensile loading while it was reduced under compressive loading for both Mg/ZrO_2_ and Mg/(ZrO_2_ + Cu) composites.

## 1. Introduction

Magnesium is the lightest metallic construction material which is ~35% lighter than aluminum. Owing to its low density, magnesium offers high specific mechanical properties. In addition, magnesium has other favorable advantages including high damping capacity, high dimensional stability, good machinability, good electromagnetic shielding characteristics and recyclability. With these beneficial properties, magnesium becomes an attractive material for manufacturing lighter components/products for diverse applications. In practical and commercial applications, magnesium is mostly used in the form of alloys [1,2]. With the advent of composite technology, researchers have also made extensive studies on the development of high performance magnesium composites. Magnesium composites that were synthesized mostly contained micron-sized particles comprising of ceramic reinforcements such as carbides, oxides, nitrides and borides and metallic reinforcement such as Ti, Cu and Ni [3,4,5,6]. While the strengths in magnesium composites can be improved using micron particles, the ductility was inevitably decreased. Previous studies [7,8,9,10,11] on magnesium nanocomposites showed the ability of nano particulate reinforcements on enhancing strength and/or ductility of magnesium. Commonly, the nanocomposites are synthesized using single ceramic [7,8,9] or metal reinforcements [10,11]. Reviews of work on particle reinforced magnesium based nanocomposites can be found in recent publications by Dieringa [12] and Ferguson *et al*. [13]. In addition, recent investigations were also made on the magnesium composites containing hybrid particle reinforcements [14,15,16,17,18,19,20,21]. Hybrid reinforcements were prepared by using different combinations such as “ceramic + ceramic”, “ceramic + CNT” and “ceramic + metal” in Mg matrix. Among those combinations, as reported in the previous investigations [12,13,14,15,16,17,18,19,20,21], “ceramic + metal” hybrid reinforcements in Mg matrix offered the best improvement in mechanical properties in the related composite systems.

Accordingly, the present study focused on the synthesis of magnesium composites using single (ZrO_2_) and hybrid (ZrO_2_ + Cu) reinforcements in nano length scale. The aim is to investigate the effect of addition of nano ZrO_2_ and different combination of hybrid reinforcements on the mechanical properties of pure magnesium. Materials synthesis was carried out using the microwave assisted powder metallurgy route. Characterizations on microstructure, hardness, tensile and compressive properties were done on the extruded samples. Particular emphasis was placed to study the effect of single and hybrid reinforcements on the variation in microstructure and mechanical properties of magnesium. Furthermore, the use of different extrusion ratio on the properties of Mg/ZrO_2_ composite was also investigated.

## 2. Results and Discussion

### 2.1. Grain Size and Reinforcement Distribution

**Figure 1:**
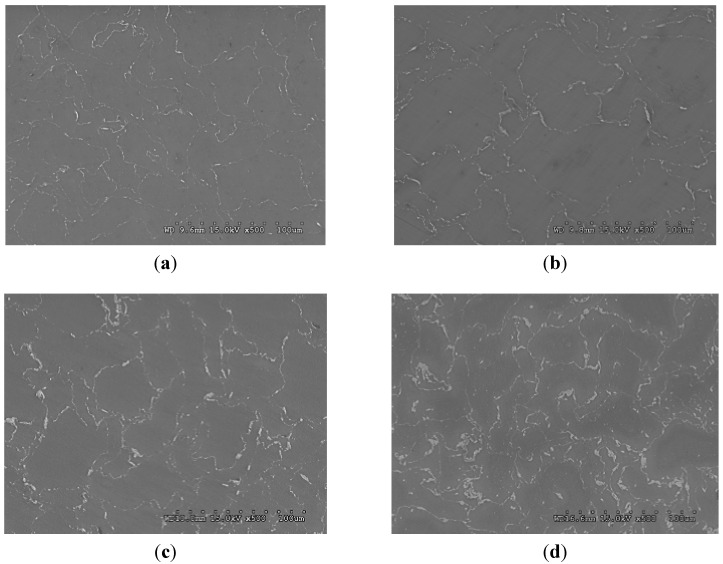
Field Emission Scanning Electron Microscope (FESEM) micrographs showing second phase distribution in: (**a**) Mg/0.3ZrO_2_; (**b**) Mg/1.0ZrO_2_ (20.25:1); (**c**) Mg/1.0ZrO_2_ (26:1); and (**d**) Mg/(0.3ZrO_2_ + 0.7Cu) composites.

**Figure 2:**
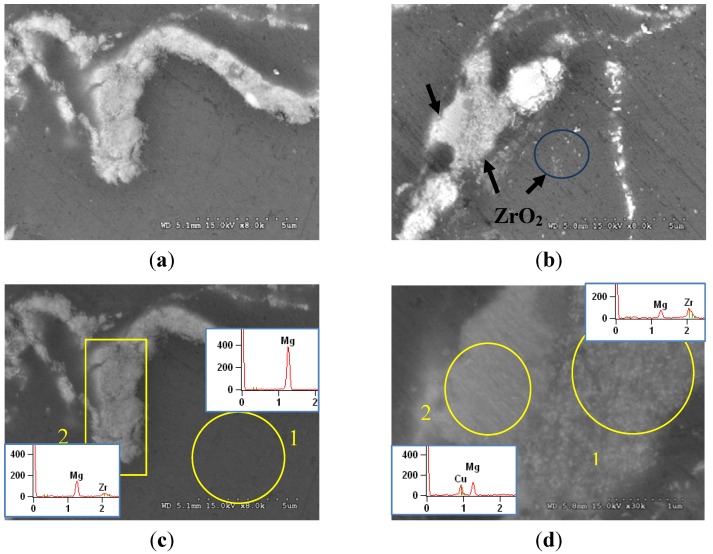
Representative micrographs showing: (**a**) clustered/agglomerated ZrO_2_ reinforcements in Mg/1ZrO_2_ composite, (**b**) the presence of Cu, clustered and dispersed ZrO_2_ phases in Mg/(0.6ZrO_2_ + 0.4Cu) hybrid composite and corresponding energy dispersive X-ray spectroscopy (EDS) analysis in: (**c**) and (**d**).

Grain size measurement results from Table 1 show that there was no significant change in grain size when ZrO_2_ reinforcement was present in Mg matrix regardless of the amount of the reinforcement being added. It was realized that, especially for fine particle reinforced composites, grain refinement can only be achieved by using a sufficiently high amount of the reinforcement particles in the matrix material [22]. However, it can be noticed from the current results that matrix grain refinement not only depends on the amount of the reinforcement but also on the particle distribution (Table 1 and Figure 1). When the ZrO_2_ content increased from 0.3 to 1.0 vol % in Mg matrix, the reinforcing particles tend to form larger clusters/agglomerates maintaining the same distribution pattern without dispersing them throughout the matrix (Figure 1a,b). This indicates that the uniformity of particle distribution is insufficient to provide the grain size reduction in all Mg/ZrO_2_ compositions. Consequently, the grain size in Mg/ZrO_2_ composites remained the same when compared to that of Mg. Also, applying high extrusion ratio (from 20.25:1 to 26:1) has no effect on the grain size variation showing the similar grain size in case of Mg/1.0ZrO_2_ composite. But the use of increased extrusion ratio provided better reinforcement distribution as indicated by the smaller space between the clustered nanoparticles shown in Figure 1c. In case of Mg/(ZrO_2_ + Cu) hybrid composites, a significant reduction in grain size was observed. The reduction was about one third when compared to that of pure Mg and Mg/ZrO_2_ composites. This indicates the usefulness of copper as hybrid reinforcement assisting in the matrix grain refinement. Having limited solid solubility in magnesium, the presence of copper causes Mg_2_Cu intermetallics formation [10,16,23]. The existence of more obstacles (ZrO_2_, Cu and Mg_2_Cu intermetallics) can effectively pin the grain boundary which contributes to the grain size reduction in the hybrid composites. Unlike Mg/ZrO_2_ composite, reinforcement distribution was globally finer with increasing presence of second phases in Mg/(ZrO_2_ + Cu) composites as observed in the micrograph (Figure 1). The morphology of the Mg/ZrO_2_ material shows a more or less continuous film of clustered/agglomerated ZrO_2_ nanoparticles at the grain boundaries, whereas in the Cu-containing composite the ZrO_2_ film is discontinuous and punctuated with Cu particles. In case of Mg/ZrO_2_ composite, the ZrO_2_ particles were mostly in the clustered/agglomerated form with minimal particle dispersion within the matrix (Figure 2a).

**Table 1:** Results of grain size and hardness measurements.

Materials (vol %)	Grain size	Macrohardness	Microhardness
	(µm)	(HR15T)	(HV)
Mg	25 ± 7	44.7 ± 1.0	42.0 ± 1.6
Mg/0.3ZrO_2_	24 ± 7	46.3 ± 0.9	40.0 ± 1.0
Mg/0.6ZrO_2_	29 ± 3	46.0 ± 2.0	41.6 ± 2.1
Mg/1.0ZrO_2_	25 ± 4	44.1 ± 0.7	42.1 ± 1.9
Mg/1.0ZrO_2_ *	23 ± 6	41.8 ± 0.8	39.9 ± 1.4
Mg/(0.3ZrO_2_ + 0.7Cu)	9 ± 2	57.9 ± 1.3	47.6 ± 1.0
Mg/(0.6ZrO_2_ + 0.4Cu)	11 ± 3	61.1 ± 0.6	50.2 ± 0.9

* Extrusion ratio of 26:1 was used for this composite.

### 2.2. XRD Analysis

**Figure 3:**
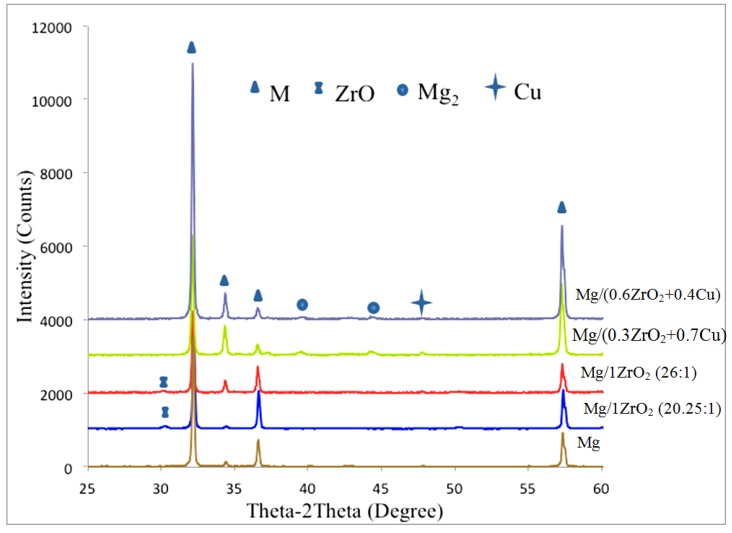
X-ray diffractograms of Mg and Mg composites.

Figure 3 shows the X-ray diffraction patterns of Mg and Mg composites. In case of Mg/1.0ZrO_2_ composites, an additional peak of ZrO_2_ was revealed when compared to Mg and Mg/(ZrO_2_ + Cu) composites. In case of Mg/(ZrO_2_ + Cu) composites, the peak related to ZrO_2_ was not present. However, the peaks matching to Cu and Mg_2_Cu were found in the XRD patterns. From the related studies [9,24], the peaks related to the ceramic reinforcement particles which are in nano length scale do not generally appear in the composites. This is due to either the amount of reinforcement being too small (less than 2 vol %) to be detected by the XRD diffractometer or the presence of fine reinforcement particles which are individually or uniformly distributed with small clusters in the matrix [25,26]. It has also been reported in previous studies [25,26] that peaks are normally detectable for the small additions of micron-sized reinforcement particles. It may thus be concluded from the current study that the appearance of ZrO_2_ peak in the Mg/1.0ZrO_2_ composites is due to the clustered/agglomerated particles as observed in the micrographs (Figure 2a,c). In Mg/(ZrO_2_ + Cu) composites, clustering tendency of ZrO_2_ reinforcement was less as discussed in the above section (Section 3.1). The evidence of smaller ZrO_2_ clusters and dispersed ZrO_2_ particles can also be seen in the micrograph (Figure 2b) which again supports the reason for the absence of ZrO_2_ peak in Mg/(ZrO_2_ + Cu) composites. On the other hand, the presence of agglomerated Cu and Mg_2_Cu phase was confirmed by XRD analysis (Figure 3) and EDS analysis (Figure 2d).

### 2.3. Hardness

Hardness measurement results in Table 1 show a minimal change in both macro- and micro- hardness values in the case of pure Mg and Mg/ZrO_2_ composites. Results suggest that the presence of single ZrO_2_ reinforcement is ineffective in increasing hardness of Mg matrix. This may primarily be attributed to poor reinforcement distribution of ceramic reinforcements. To achieve high hardness level in the composites, clustering of second phases is not preferable due to a number of facts including larger intercluster spacing and the weak bonding among brittle ceramic particulates at the clustered particles region. No variation in hardness can also be attributed to the similar grain size between Mg and Mg/ZrO_2_ composites. On the other hand, an improvement in both macro- and micro- hardness was observed in the hybrid composites when compared to both Mg and Mg/ZrO_2_ composites. This may primarily be attributed to the grain refinement resulting from the distribution of second phases (Table 1 and Figure 1). The presence of hard Mg_2_Cu intermetallics can additionally provide an increase in hardness level of Mg matrix [27].

### 2.4. Tensile Properties

**Figure 4:**
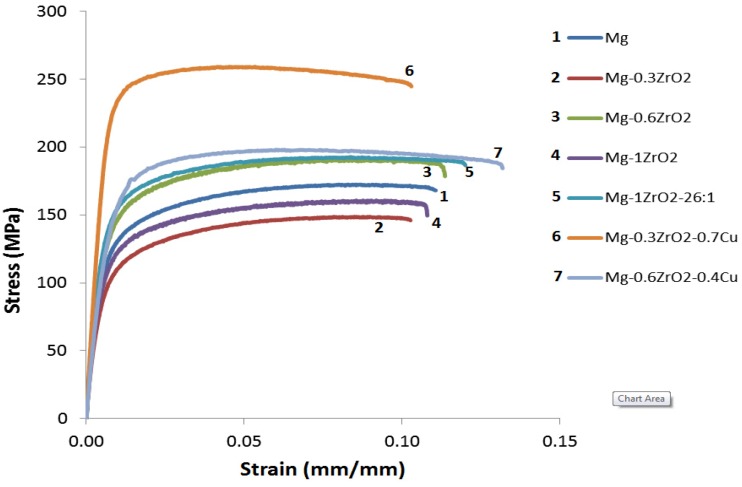
Representative stress-strain curves of Mg and Mg composites.

The results of room temperature tensile properties are shown in Figure 4 and summarized in Table 2. Not only the presence but also the increasing amount of ZrO_2_ reinforcement shows no effect on the variation of 0.2% yield and ultimate tensile strengths showing similar strength levels of pure Mg and composite samples. In fact, the strengths in Mg/0.3ZrO_2_ and Mg/1.0ZrO_2_ composites tend to decrease when compared to pure magnesium. Presence of clustered ZrO_2_ particulates in Mg matrix (Figure 1a,b and Figure 2a) could be the prime reason for this decrement. The reduction in strengths originates from weak adhesion among ceramic particulates within clusters and/or between matrix and clustered particulates [4,28]. This also indicates the lack of load transfer from the matrix to ceramic reinforcement clusters and thus yielding in the Mg/ZrO_2_ composites follows the matrix yielding behavior that contains defects. The use of high extrusion ratio (20.25:1 to 26:1) provides improvement in both 0.2% yield and ultimate tensile strengths to some extent. This is in line with the improved reinforcement distribution in Mg/1.0ZrO_2_ composite with the use of higher extrusion ratio, 26:1 (Figure 1c). However, a significant strength increment was not achieved due to partial break down of reinforcement clusters [29] and lack of grain refinement after applying high extrusion ratio (26:1) in Mg/1.0ZrO_2_ composition. Expecting to acquire better tensile properties, Cu was added as hybrid reinforcement to Mg/ZrO_2_ compositions. To fix the total reinforcement amount to be 1 vol %, 0.7 vol % and 0.4 vol % Cu were added to the Mg/0.3 vol % ZrO_2_ and Mg/0.6 vol % ZrO_2_ compositions, respectively. From the tensile test results (Table 2), a significant improvement in both 0.2% yield and ultimate tensile strengths were observed in the hybrid composites. The improvement in strengths can mainly be attributed to: (a) the combined presence of ZrO_2_, Cu and additional Mg_2_Cu intermetallics; (b) effective load transfer from matrix to the reinforcements/second phases conforming uniform reinforcement/second phase distribution; (c) grain refinement as indicated earlier in Table 1. The reduction in grain size is primarily responsible for strength improvement in magnesium hybrid composites. Additionally, the strength increment may be supported by the effective load transfer mechanism. Irrespective of enhanced tensile strengths shown by the hybrid composites, failure strain is similar to that of pure Mg and Mg/ZrO_2_ composites.

**Table 2:** Results of tensile properties.

Materials (vol %)	0.2% YS (MPa)	UTS (MPa)	Failure Strain (%)
Mg	111 ± 7.8	177 ± 10	9.0 ± 2.2
Mg/0.3ZrO_2_	84.8 ± 8.0	139 ± 7.5	8.1 ± 1.6
Mg/0.6ZrO_2_	117 ± 11	182 ± 14	9.4 ± 2.7
Mg/1.0ZrO_2_	97.8 ± 6.3	158 ± 12	8.6 ± 2.2
Mg/1.0ZrO_2_ *	122 ± 7.7	188 ± 5.9	10 ± 1.3
Mg/(0.3ZrO_2_ + 0.7Cu)	196 ± 16	249 ± 7.5	8.2 ± 1.1
Mg/(0.6ZrO_2_ + 0.4Cu)	139 ± 22	193 ± 21	11.4 ± 2.9

* Extrusion ratio of 26:1 was used for this composite.

### 2.5. Tensile Fractography

**Figure 5:**
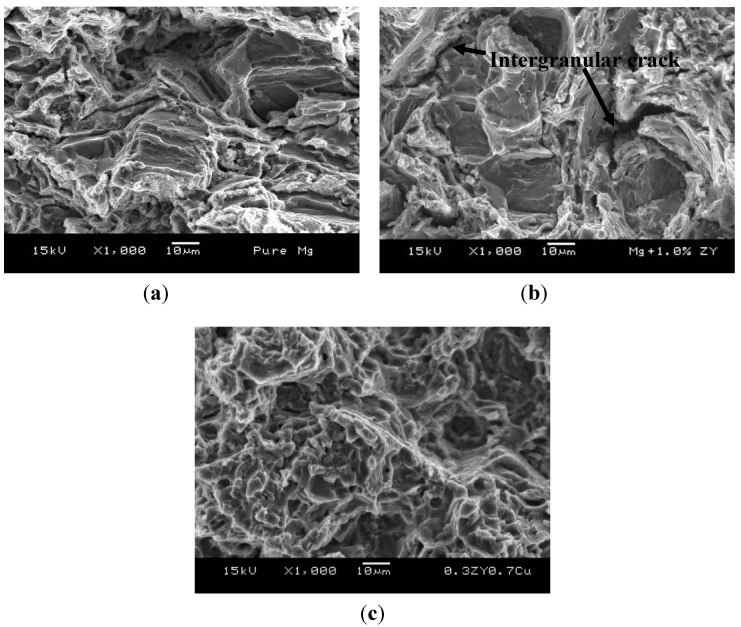
Representative tensile fracture features in: (**a**) pure Mg; (**b**) Mg/1.0ZrO_2_ (20.25:1) composite; and (**c**) Mg/(0.3ZrO_2_ + 0.7Cu) hybrid composite.

Tensile fractographs can be seen in Figure 5. From the failure analysis, the same fracture features revealing rough fracture surfaces and cleavage fracture were observed in pure Mg and Mg/ZrO_2_ composites. Owing to HCP crystal structure, magnesium’s deformability is limited due to the lack of sufficient slip activity. Although the size of clustered reinforcements was in micron length scale, premature failure was not observed in the Mg/ZrO_2_ composites observing similar failure strain when compared to magnesium (Table 2). This is different from micron size particle reinforced magnesium composites in which failure strain reduction was commonly found when compared to that of unreinforced magnesium. The premature failure in these composites is mainly due to the debonding between the matrix and reinforcement particles, and rapid particle cracking [4]. In the current study, there were two possibilities for Mg/ZrO_2_ composite eventual failure: (a) the matrix failure could control the fracture behavior in the composites observing the similar fracture features and (b) failure due to intergranular cracking initiated from micro-cracking of the reinforcement clusters at the grain boundary (Figure 5b). In case of hybrid composite, fine and homogenous fracture features were observed (Figure 5c). This implies the achievement of high strength in hybrid composites yet the failure strain was not improved (Table 2). This can be primarily attributed to the increasing presence of brittle second phases and the clustered ones remained at the grain boundary.

### 2.6. Compressive Properties

Table 3 shows the room temperature compressive properties. From the results, the same compressive yield strength was observed between pure Mg and Mg/ZrO_2_ composites while average ultimate compressive strength was marginally reduced in case of Mg/ZrO_2_ composites when compared to pure Mg. It is well known that yielding in magnesium materials is mainly due to twinning. Compressive yield strength of magnesium can commonly be increased by reducing the twinning activity through grain refinement [30,31,32]. In the present study, having similar grain size, compressive yield strength was not improved in Mg/ZrO_2_ composites when compared to pure Mg. It also indicates that there was no strengthening effect from the ZrO_2_ reinforcement. In case of hybrid composite, both compressive yield and ultimate compressive strengths are significantly increased when compared to Mg and Mg/ZrO_2_ composites. This can be mainly attributed to the grain refinement observed in Mg/(ZrO_2_ + Cu) composites. For the failure strain in the composites, it was reduced when compared to pure Mg. It suggests that the presence of reinforcement either in the form of single (ZrO_2_) or hybrid (ZrO_2_ + Cu) can deteriorate the compressive failure strain of magnesium. From the related investigations [33,34], it was also found that the compressive failure strain decreased in magnesium composites when compared to its unreinforced counterpart. The resultant failure strain reduction in the current composites is fundamentally in agreement with the observations from the previous studies.

**Table 3:** Results of compressive properties.

Materials * (vol %)	0.2% YS (MPa)	UTS (MPa)	Failure Strain (%)
Mg	109 ± 4	284 ± 11	23 ± 3
Mg/0.3ZrO_2_	109 ± 6	273 ± 13	19 ± 1
Mg/1.0ZrO_2_	109 ± 5	262 ± 18	19 ± 4
Mg/(0.3 ZrO_2_ + 0.7Cu)	124 ± 7	352 ± 18	12 ± 3

* Extrusion ratio of 20.25:1 was used for all materials.

### 2.7. Compressive Fractography

**Figure 6:**
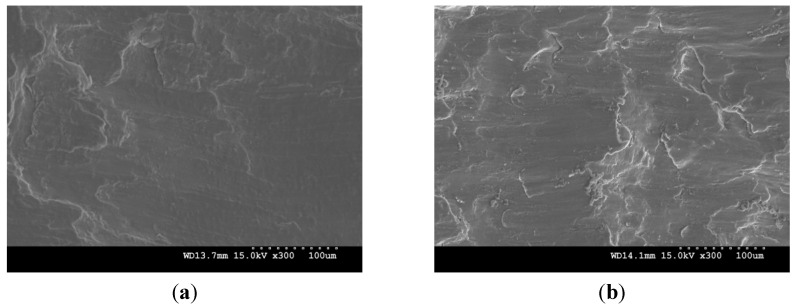

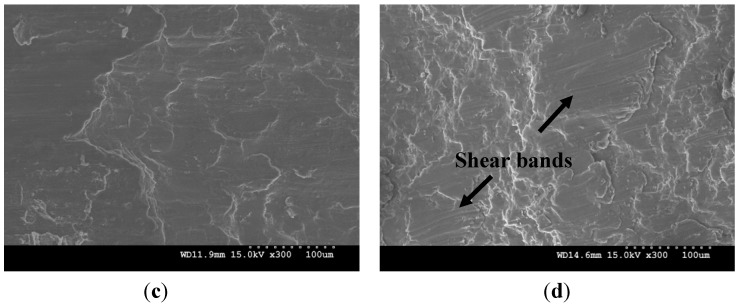
Representative compressive fractographs of: (**a**) pure Mg; (**b**) Mg/0.3ZrO_2_; (**c**) Mg/1.0ZrO_2_ and (**d**) Mg/(0.3ZrO_2_ + 0.7Cu) composites.

Compressive fractographs are shown in Figure 6. In case of pure magnesium and Mg/ZrO_2_ composites (Figure 6a–c), the fracture surfaces are relatively smooth and the shear band formation can hardly be seen in the failed samples. According to the investigation from Ion *et al.* [35] on single phase Mg alloy, shear band formation was due to heterogeneous deformation in materials arising from localized deformation at grain boundary leaving cores of the grains undeformed under compressive loading. The minimal presence of shear bands in pure Mg indicates that there was homogenous deformation due to the presence of fewer obstacles through less grain boundary area and absence of secondary phases. This deformation mechanism can also be applied to the Mg/ZrO_2_ composites having similar grain size and minimal or the lack of dispersion of ZrO_2_ particles within the grain interior (Figure 2a,c). In contrast, heterogeneous fracture surface and the presence of shear bands are found in Mg/(ZrO_2_+Cu) hybrid composite (Figure 6d). The plastic deformation in the hybrid composite was constrained due to the presence of dispersed second phases and comparatively large amount of grain boundaries. This led to the significant reduction in compressive failure strain in the hybrid composite (Table 3).

## 3. Experimental Section

### 3.1. Materials

The material used for the matrix is magnesium (Mg) powder of 98.5% purity with particle sizes ranging from 60 to 300 μm (Merck, Germany). As for the reinforcements, yttria-stabilized Zirconia (ZrO_2_) of 99.9% purity with particle sizes ranging from 51 to 65 nm and copper (Cu) of 99.8% purity with an average particle size of 25 nm (Nanostructured & Amorphous Materials, Inc., TX, USA) were used.

### 3.2. Processing

The primary processing for the composites was done in sequential order of blending, pressing and sintering according to the powder metallurgy technique. Magnesium and reinforcement powders were first weighed in accordance with the designated compositions; Mg/0.3 vol % ZrO_2,_ Mg/0.6 vol % ZrO_2,_ Mg/1.0 vol % ZrO_2_, Mg/(0.3 vol % ZrO_2_ + 0.7 vol % Cu) and Mg/(0.6 vol % ZrO_2_ + 0.4 vol % Cu). The powder mixture was then blended using planetary ball milling machine at 200 rpm for 1 h followed by cold compaction. No balls or process control agent was used during the blending step. Compaction was done at a pressure of 97 bars (510 MPa) to form billets of 35 mm diameter and 40 mm height using a 100 ton press. Monolithic magnesium was compacted using the same parameters but without blending. The compacted billets were sintered using a hybrid microwave sintering technique to a temperature near the melting point of magnesium (~640 °C) in a 900 W, 2.45 GHz SHARP microwave oven. Microwave sintering was carried out in ambient atmospheric conditions.

The secondary processing involved the hot extrusion of the sintered billets. The sintered billets were subsequently hot extruded at a temperature of 350 °C at an extrusion ratio of 20.25:1 to produce 8 mm diameter extruded rods. During extrusion of Mg/1.0 vol % ZrO_2_ composition, two different extrusion ratios, 20.25:1 and 26:1 were used. Prior to extrusion, the sintered billets were soaked in a resistance furnace at a temperature of 400 °C for 1 h.

### 3.3. Microstructure Characterization

Microstructural characterization studies were conducted to determine the grain size and distribution of reinforcements. OLYMPUS metallographic optical microscope, Scion Image Analyzer and HITACHI S-4300 Field Emission Scanning Electron Microscope (FESEM) equipped with energy dispersive X-ray spectroscopy (EDS) were used for this purpose.

### 3.4. X-ray Diffraction Studies

X-ray diffraction analysis was carried out on the polished extruded Mg and Mg composite samples using automated Shimadzu LAB-X XRD-6000 diffractometer. The samples were exposed to CuK_α_ radiation (λ = 1.54056 Å) at a scanning speed of 2 °C/min. The Bragg angle and the values of the interplanar spacing (d) obtained were subsequently matched with the standard values for Mg, ZrO_2_, Cu and related phases.

### 3.5. Mechanical Testing

The mechanical behavior of both monolithic and composite samples was quantified in terms of hardness, tensile and compressive properties. Macrohardness measurements were made using Future-Tech FR-3 Rockwell Type Hardness Tester. The test was conducted under conditions of 2 s dwell time and a test load of 15 kgf using steel ball indenter (1.588 mm) in accordance with ASTM E18-02. Microhardness measurements were performed on the magnesium matrix of the polished samples using Shimadzu-HMV automatic digital microhardness tester. The test was done using a Vickers indenter under a test load of 25 gf and a dwell time of 15 s in accordance with the ASTM standard E384-99.

The tensile properties of the as-extruded monolithic magnesium and its composite counterparts were determined in accordance with procedures outlined in ASTM standard E8M-01. The tensile tests were conducted on round tension test specimens (5-mm gage diameter and 25-mm gage length) on MTS 810 automated servo-hydraulic mechanical testing machine at a crosshead speed set at 0.254 mm/min.

Compression tests were performed on cylindrical monolithic and composite samples according to ASTM E9-89a using MTS 810 automated servo-hydraulic mechanical testing. Extruded rod of 8 mm diameter was cut into 8 mm length samples for compression tests to provide the aspect ratio (l/d) of unity. Samples were tested at a strain rate of 5 × 10^−3^ min^−1^ and the compression load was applied parallel to the extrusion direction.

### 3.6. Fracture Behavior

To investigate the failure mechanism during tensile and compressive loadings, the tensile and compressive fractured surfaces of pure Mg and its composite specimens were characterized using scanning electron microscope (SEM).

## 4. Conclusions

The major conclusions are as follows:

1. Magnesium based nanocomposites and hybrid composites can be successfully synthesized using microwave sintering assisted powder metallurgy approach.
2. The formation of ZrO_2_ reinforcement clusters in Mg matrix resulted in the lack of grain refinement in all Mg/ZrO_2_ composite compositions. On the other hand, the use of hybrid reinforcements (ZrO_2_ + Cu) realized significant grain size reduction in magnesium hybrid composites.
3. Both macro- and micro-hardness, tensile, and compressive strengths were improved only in the Mg/(ZrO_2_ + Cu) hybrid composites primarily as a result of their finer grain size.
4. Tensile failure strain remained the same whereas compressive failure strain was decreased in all composites when compared to pure Mg.
5. Tensile failure analysis revealed the similar rough fracture features in the case of Mg and Mg/ZrO_2_ composites. Refined fracture features were observed in the hybrid composite.
6. Under compressive loading, the presence of shear bands was minimally observed from the fracture surfaces of Mg and Mg/ZrO_2_ composites. In case of hybrid composite, a large amount of shear band formation was observed.
